# *Sarcocystis* sp. infection associated to encephalomyelitis and myositis in a Brazilian dog

**DOI:** 10.1007/s11259-026-11598-2

**Published:** 2026-10-31

**Authors:** Ítalo Henrique do Nascimento Iara, Ana Flávia Goveia Martins, Luana de Sousa Ribeiro, Fabiana Marques Boabaid, Luiz Gustavo Schneider de Oliveira, Emanoelly Machado Sousa da Silva, David Driemeier, Bruno Benetti Junta Torres

**Affiliations:** 1 Escola de Veterinária e Zootecnia, Universidade Federal de Goiás, Goiânia, Goiás Brasil; 2 Instituto de Ciências Agrárias, Universidade Federal dos Vales do Jequitinhonha e Mucuri (UFVJM), Unaí, Minas Gerais Brasil; 3 College of Veterinary Medicine, Iowa State University, Ames, IA USA; 4 Faculdade de Veterinária, Universidade Federal do Rio Grande do Sul, Porto Alegre, Rio Grande do Sul Brasil

**Keywords:** Canine, Immunohistochemistry, Meningoencephalomyelitis, Protozoa, South America, Apicomplexa

## Abstract

*Sarcocystis* sp.-induced disease is known especially in horses, in which *S. neurona* is the primary causative agent of Equine Protozoal Myeloencephalitis in the Americas. While horses are aberrant hosts, the range of susceptible species includes marine mammals, raccoons, and cats. Reports of clinical disease in dogs are rare, remaining unreported in South America. This study describes the clinical, pathological, and immunohistochemical findings of a naturally occurring, fatal case of meningoencephalomyelitis and myositis caused by *Sarcocystis* sp. in a dog in Brazil. A 5-year-old mixed-breed bitch presented with acute non-ambulatory tetraparesis, generalized muscle atrophy, and blindness. Despite supportive therapy, the patient deteriorated and was euthanized. Gross findings included enlarged lymph nodes, moderate splenomegaly and hepatomegaly, with multifocal mild fibrin deposition on the liver capsule, accompanied by multifocal mild hemorrhages in the pancreas. Histopathology demonstrated severe, multifocal necrotizing meningoencephalomyelitis, myositis, splenitis, lymphadenitis and interstitial pneumonia, associated with intralesional protozoal schizonts. Immunohistochemistry supported immunolabelling consistent with *Sarcocystis* sp. while excluding *Toxoplasma gondii* and *Neospora caninum*. To the authors’ knowledge, this represents the first report of clinical canine sarcocystosis with this severity in South America supported by histopathological and immunohistochemical findings. These findings support that *Sarcocystis* sp. should be considered a differential diagnosis for canine progressive neurological disease, highlighting the potential role of wildlife reservoirs in the disease’s epidemiology.

## Background

*Sarcocystis* is a genus of apicomplexan parasites that includes *S. neurona*, the primary etiological agent of equine protozoal meningoencephalitis (EPM) in the Americas (de Lahunta et al. 2021; Dubey et al. 2015). Although opossums (*Didelphis* spp.) are the definitive hosts, the parasite can infect a wide range of intermediate and aberrant hosts, including sea otters, raccoons, cats, and rarely, dogs (Abdolahi et al. 2025; Dubey et al. 2000; Gondim et al. 2021; Nawshirwan et al. 2023; Rosenthal 2021).

In dogs, *Sarcocystis-*related disease is rarely reported (Allison et al. 2006; Dubey et al. 1991, 2006, 2014; Gerhold et al. 2014; Sykes et al. 2011; Trasti et al. 1999). However, it can manifest as severe multifocal neurological deficits, systemic inflammation, or a combination of both (Dubey et al. 2014; Irvine et al. 2016). The scarcity of documented clinical cases has limited the understanding of its pathogenicity and epidemiological significance in canine, especially in South America where the definitive host is prevalent (Gondim et al. 2021).

This report aims to describe the clinical, anatomopathological, and immunohistochemical features of a naturally acquired, fatal infection associated with *Sarcocystis* sp. in a dog in Brazil. We highlight the diagnostic challenges of protozoal encephalitis, the dissemination to multiple organ systems, and the importance of differentiating this agent from other neurotropic protozoa.

## Case presentation

A 5-year-old, intact female mixed-breed dog was presented to the Neurology and Neurosurgery Service of a Veterinary Teaching Hospital with a six-day history of lateral recumbency and acute vision loss. The patient lived on a rural property with livestock and other healthy dogs. Vaccination and deworming protocols were not up to date.

Physical examination revealed a body condition score of 5/9, mild dehydration, and diffuse muscle atrophy, which was more pronounced in the pelvic limbs. Ophthalmic inspection showed bilateral cataracts and corneal opacity. Orthopedic palpation elicited pain in the left thoracic limb, where soft tissue swelling was noted around the elbow.

Neurological examination demonstrated a depressed mental status, non-ambulatory tetraparesis, and focal myoclonus of the right temporal muscle. Cranial nerve assessment revealed a reduced gag reflex and bilateral absence of menace response. Spinal reflexes and muscle tone were reduced in all four limbs. Neuroanatomic localization was multifocal, involving the forebrain, brainstem, and spinal cord segments (C6-T2 and L4-S3), with a peripheral nervous system component.

Initial diagnostic workup included a complete blood count (CBC), serum biochemistry (creatinine and alanine aminotransferase [ALT]), abdominal ultrasonography, and a distemper antigen immunochromatography test (urine). CBC revealed absolute neutrophilia (14,863/mm³; RI: 3,000–11,500/mm³) with normal leukocyte counts, mild anisocytosis, and rouleaux formation. Serum biochemistry was unremarkable. The distemper antigen test was negative. Abdominal ultrasonography revealed hepatomegaly, splenic nodular hyperplasia, and enlarged aortic-lumbar lymph nodes with degenerative echotexture. Radiographs of the left forelimb showed soft tissue swelling consistent with inflammation. Magnetic resonance imaging (MRI) and cerebrospinal fluid (CSF) analysis were recommended but were declined by the owner due to financial constraints and the patient’s unstable condition.

The patient was hospitalized for supportive care, receiving fluid therapy (Lactated Ringer’s solution), analgesia (methadone 0.3 mg/kg IM q6h; metamizole 25 mg/kg IV q8h), and antibiotic therapy (ampicillin 20 mg/kg IV q8h). Due to a lack of improvement after 48 h, the antibiotic protocol was escalated to ampicillin-sulbactam (20 mg/kg IV q8h) and tramadol (4 mg/kg IM q8h) was added to adjusted analgesia. Despite intensive care, the patient’s neurological status progressively deteriorated. The owner elected for humane euthanasia, and a full necropsy was authorized.

Gross examination revealed pale mucous membranes. The right axillary lymph node was enlarged and firm. The liver was enlarged with rounded edges, evidenced lobular pattern, multifocal petechiae, and mild fibrin deposition on the capsule. Lymphadenomegaly was observed affecting hepatic, gastric, and mesenteric nodes. The spleen was moderately enlarged with diffuse white pulp hyperplasia. The lungs showed multifocal reddish areas in the caudal lobes and frothy exudate in the airways. During necropsy the tissues were collected in 10% neutral buffered formalin, routinely processed for histopathology and stained with hematoxylin and eosin (HE).

**Fig. 1:**
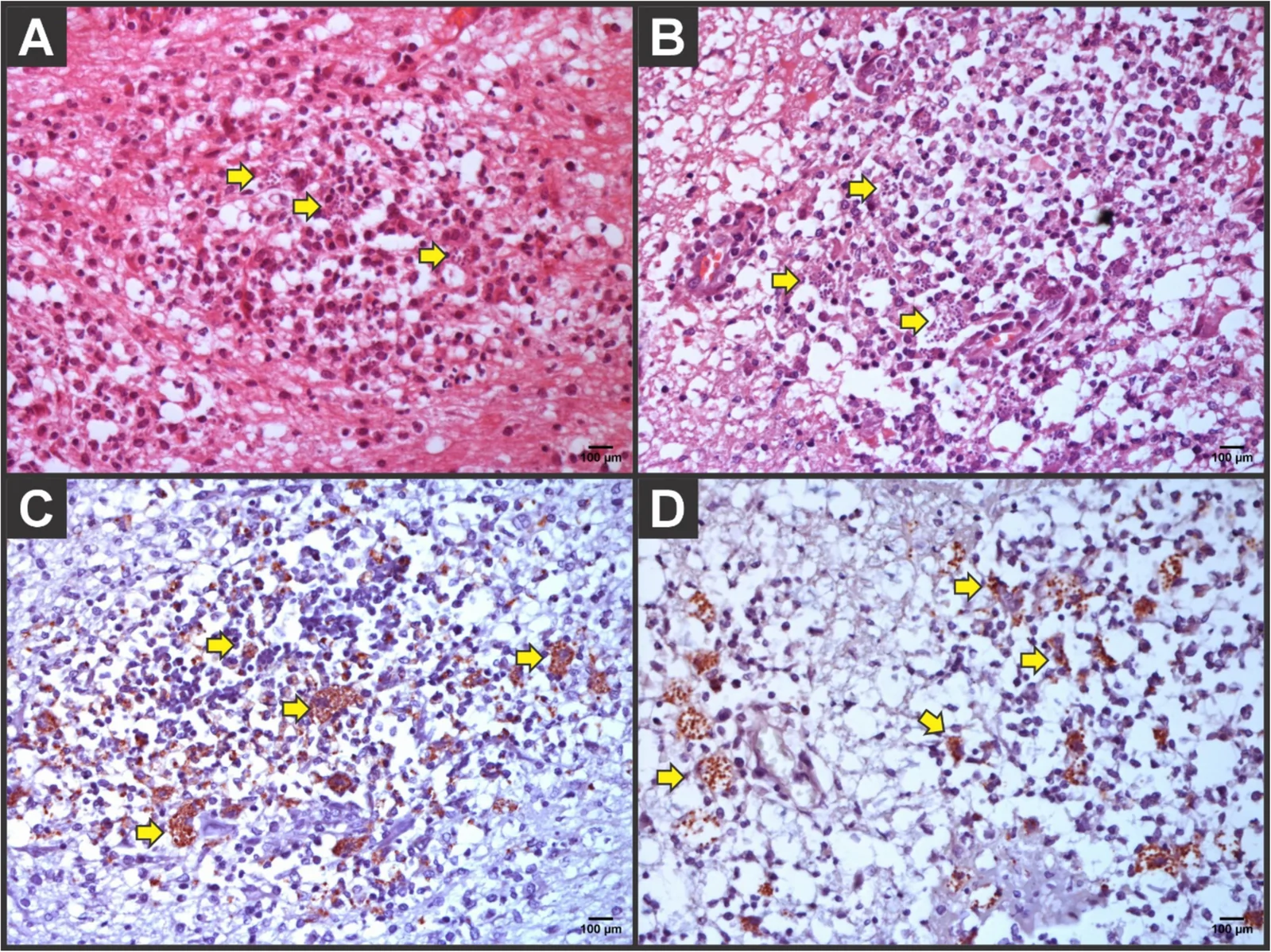
Histopathology and immunochemistry (IHC) slides of canine sarcocystosis in central nervous system. A: Brain, numerous protozoal structures (arrows) intermingled in an area of necrosis, with multifocal severe inflammatory infiltrate composed of macrophages, neutrophils, lymphocytes and plasma cells. (HE, obj. 40x). B: Spinal cord, thoracic segment, schizonts containing basophilic merozoites (arrows) within a focal area of necrosis and severe mixed inflammatory infiltrate (HE, obj. 40x); C: Brain, strong positive immunolabeling for *Sarcocystis* sp. (arrows) (polyclonal anti-*Sarcocystis neurona*, IHC, AEC chromogen 40x). D: Spinal cord, strong positive immunolabeling for *Sarcocystis* sp. (arrows) (polyclonal anti-*Sarcocystis neurona*, IHC, AEC chromogen 40x)

Histopathologic evaluation of the brain, including the frontal, parietal and occipital cortex, pons, cerebellum, and sections of the cervical, thoracic, lumbar and sacral spinal cord revealed randomly distributed multifocal areas of liquefactive necrosis, compromising up to 50% of the parenchyma in the pons and significant portions of the cortex and spinal cord (Fig. 1). Intermingled in areas of necrosis was a severe inflammatory infiltrate of macrophages, lymphocytes, plasma cells and neutrophils, with multifocal moderate gliosis and edema. Similar inflammatory infiltrate was seen expanding the perivascular spaces and meninges. Within the necrotic foci were numerous protozoan structures characterized as thin-walled schizonts, up to 20 μm in diameter, containing numerous oval basophilic merozoites, measuring 2 × 3 μm (Fig. 1). Merozoites were also found free within areas of necrosis. Skeletal muscles and tongue exhibited lymphoplasmacytic and histiocytic myositis with myofiber necrosis and intralesional protozoa.

The protozoal structures were also identified in the spleen, lymph nodes and lungs, accompanied by multifocal severe histiocytic splenitis and lymphadenitis, as well as multifocal moderate interstitial pneumonia. Additionally, the liver presented multifocal moderate centrilobular macrovacuolar degeneration and multifocal mild haemorrhage and the kidney exhibited multifocal moderate membranoproliferative glomerulonephritis.

Sections of the brain and spinal cord were subjected to immunohistochemistry (IHC) testing for *Toxoplasma gondii*, *Neospora caninum*, and *Sarcocystis* sp. as previously described (Henker et al. 2020). The anti- *Sarcocystis neurona* antibody was a non-commercial rabbit polyclonal primary antibody, provided by JP Dubey (Beltsville, MD, USA), produced according to the protocol by Hamir et al. (1993). Intralesional parasites showed intense positive immunoreactivity for *Sarcocystis* sp. (Fig. 1) and were consistently negative for *T. gondii* and *N. caninum*, supporting the diagnosis.

## Discussion

This report describes a rare case of fatal canine meningoencephalomyelitis and myositis associated with *Sarcocystis* sp. infection. To the authors’ knowledge, this represents the first reported description supported by histopathology and IHC of such severe systemic and neurological sarcocystosis in a dog in Latin America.

The clinical presentation of non-ambulatory tetraparesis and multifocal neurological deficits overlaps significantly with other common canine infectious diseases, such as distemper, leishmaniosis, neosporosis, and toxoplasmosis (Platt and Olby 2013). In this case, the rapid progression and severity of the lesions, characterized by extensive liquefactive necrosis, were remarkable. Similar histopathological patterns are well-documented in EPM (Dubey et al. 2015; Henker et al. 2020; Paixão et al. 2007; Saville et al. 2004) but are infrequently reported in dogs with this intensity (Cooley et al. 2007; Dubey et al. 2006; Gerhold et al. 2014; Trasti et al. 1999).

In dogs, meningoencephalomyelitis cases have been reported predominantly in the United States, whereas muscular or visceral manifestations have been reported in South America (Chapman et al. 2005; Cooley et al. 2007; Dubey et al. 2006, 2014; Gerhold et al. 2014; Mouser et al. 2009; Vashisht et al. 2005). In Brazil, the only previous report of meningoencephalitis associated with *Sarcocystis neurona* infection in small domestic animals involved a cat (Hammerschmitt et al. 2020), and a study using frozen brain samples from dogs with neurological signs identified a single PCR-positive case out of eleven samples (Ries et al. 2025). This apparent scarcity of cases may be attributed to underdiagnosis in the region, as confirmatory ancillary techniques for *Sarcocystis* sp. are not routinely performed in most diagnostic laboratories.

Differentiation from *N. caninum* and *T. gondii* is challenging using routine H&E staining due to morphological similarities. The employment of IHC markers combined with the morphology of the schizonts is helpful for this task (Dubey et al. 2006; Henker et al. 2020).

The main clinical manifestation associated with *Sarcocystis* sp. in South America is equine protozoal meningoencephalitis by *S. neurona*, whose epidemiology is intrinsically linked to the definitive host, *Didelphis albiventris* (Dubey et al. 2000, 2001; Gondim et al. 2021). High infection rates have been reported in Brazilian opossums (Gondim et al. 2021). The dog in this report lived on a farm with confirmed wildlife presence, suggesting environmental contamination or predation of infected intermediate hosts as likely transmission routes (Freitas et al. 2018; Ridgway 2021). Despite the most common clinical manifestations of *Sarcocystis* sp. infections being associated with the gastrointestinal or muscular systems, the combination of clinical signs, histopathological findings, IHC results, and epidemiological features supports the suspicion of *Sarcocystis neurona* infection in this case.

A limitation of this report was the inability to perform advanced *ante-mortem* diagnostics, such as MRI and CSF analysis, due to the patient’s clinical instability and financial constraints. Furthermore, molecular characterisation (PCR) was not possible as frozen or fresh tissue samples were not available and PCR from formalin fixed paraffin embedded tissue was negative, like other similar studies, probably due to DNA degradation (Henker et al. 2020). Although additional confirmatory methods, such as immunoblotting or immunofluorescence assays, could further strengthen the diagnosis, these analyses could not be performed because no additional samples were available. However, the diagnosis was achieved through the combination of characteristic histopathological findings and immunohistochemistry tests. The distinct morphology of the schizonts, coupled with positive immunoreactivity for *Sarcocystis* sp. and negative results for *T. gondii* and *N. caninum*, the primary differential diagnoses for protozoal meningoencephalomyelitis in dogs, supports the diagnosis (Dubey et al. 2015; Henker et al. 2020).

To our knowledge, this is one of the few naturally occurring cases of neurological and systemic sarcocystosis by *Sarcocystis* sp. reported in a dog, contributing valuable clinical and pathological information to the veterinary literature. This case highlights the need for veterinary clinicians in Latin America to consider *Sarcocystis* sp. in the differential diagnosis of dogs with multifocal neurological signs, especially in rural settings.

## Data Availability

No datasets were generated or analysed during the current study.
